# Eye-mediated immune tolerance to Type II collagen in arthritis-prone strains of mice

**DOI:** 10.1111/jcmm.12376

**Published:** 2014-09-11

**Authors:** Shukkur M Farooq, Ashok Kumar, Hossam M Ashour

**Affiliations:** a Department of Pharmacy Practice, Eugene Applebaum College of Pharmacy and Health Sciences, Wayne State UniversityDetroit, MI, USA; b Kresge Eye Institute/Dept. of Ophthalmology, Wayne State UniversityDetroit, MI, USA; c Dept. of Anatomy and Cell Biology, Wayne State UniversityDetroit, MI, USA; d Department of Microbiology and Immunology, Faculty of Pharmacy, Cairo UniversityCairo, Egypt

**Keywords:** ACAID, peripheral tolerance, immune privilege, regulatory T cells, collagen type II, C57BL/6 mice, DBA/1 mice

## Abstract

Type II collagen (CII) is a cartilage structural protein that plays important roles in joint function, arthritis and ageing. In studying the ability of CII to induce eye-mediated specific immune tolerance, we have recently proven that CII is capable of inducing anterior chamber-associated immune deviation (ACAID) in Balb/c mice. Here, we study the ability of CII to induce eye-mediated immune tolerance in strains of mice that are prone to the induction of rheumatoid arthritis. Thus, we hypothesized that CII induces ACAID in DBA/1 mice and in C57BL/6 mice through the AC route (direct injection) or the intravenous route (adoptive transfer of *in vitro*-generated CII-specific ACAID macrophages or of CII-specific *in vitro*-generated T regulatory cells). Specific immune tolerance induction was assessed using both delayed-type hypersensitivity (DTH) and local adoptive transfer (LAT) assays. Results indicated the ability of CII to generate CII-specific ACAID-mediated immune tolerance *in vivo* and *in vitro* in both DBA/1 mice and C57BL/6 mice. These findings could be beneficial in studies of immune tolerance induction using CII.

## Introduction

A prominent form of eye-mediated peripheral tolerance is termed anterior chamber-associated immune deviation (ACAID), which can be successfully induced in mice, primates and humans [1]. Following their entry into the immune privileged anterior chamber (AC) of the eye, antigens are regionally processed before getting picked up and processed by F4/80^+^ macrophages [2] that induce peripheral tolerance mediated *via* secretion of TGF-β in concert with the concomitant down-regulation of CD40 and interleukin-12 [3,4]. These macrophages have been shown to induce an antigen-specific state of immune tolerance by migrating directly to the spleen [5] *via* bloodstream within the trabecular meshwork [6], and with the help of splenic accessory immune cells [7,8]. This form of tolerance is mainly characterized by antigen-specific suppression of delayed-type hypersensitivity (DTH) reactions mediated through CD4^+^ afferent T regulatory cells (Tregs) and CD8^+^ efferent Tregs [9,10]. CD8^+^ Tregs have also been proven to play major suppressive roles in various autoimmune diseases.

Rheumatoid arthritis (RA) is characterized by the chronic inflammation of joints and is an autoimmune disorder which gradually erodes bone and cartilage. Type II collagen (CII)-induced arthritis has proven to be a powerful model of human RA and has helped delineate the roles of cellular and molecular mediators in the pathogenesis of inflammatory joint disease [11]. Degradation of CII has also been associated with ageing and osteoarthritis [12]. The clinical significance of CII in RA is well-established [13]. Studies reported that arthritis could be induced in C57BL/6 mice when immunized with CII [14–16]. CII-mediated ACAID induction resulting from the injection of CII into the AC of the eye or the adoptive transfer of *in vitro*-generated CII-specific ACAID antigen-presenting cells (APCs) in Balb/c mice has been recently shown by our group [17,18]. In this study, we aimed to test for CII-induced ACAID in arthritis-prone strains of mice (C57BL/6 and DBA/1). We hypothesized that CII-specific immune tolerance can be induced either through AC injection of CII or the adoptive transfer of *in vitro*-generated ACAID macrophages that are specific to CII into C57BL/6 or DBA/1 mice. We tested for ACAID generation following the AC injection of CII or intravenous injection of CII-specific ACAID APCs in C57BL/6 or DBA/1 mice by DTH assays. Further, we examined the regulatory functions of *in vitro*-generated CII-specific T cells in C57BL/6 and DBA/1 mice using local adoptive transfer (LAT) assays. This is the first study to report the *in vivo* and *in vitro* generation of CII-specific ACAID-mediated tolerance in DBA/1 and C57BL/6 mice.

## Materials and methods

### Mice

C57BL6 and DBA/1 mice (6–8 weeks of age) were procured from Jackson Laboratories (Bar Harbor, ME, USA). All animals were maintained at the animal care facility of the Eugene Applebaum College of Pharmacy and Health Sciences. Experiments were conducted in accordance to the guidelines of the Institutional Animal Care and Use Committee (IACUC), Wayne State University.

### ACAID induction *via* the AC injection of type II collagen

We used the Hamilton automatic dispensing apparatus (Hamilton, Whittier, CA, USA) to induce ACAID in C57BL6 and DBA/1 mice as described in our previous publications [7,19]. Isoflurane anaesthesia (2–3% isoflurane with oxygen supply) is used to anaesthetize C57BL6 and DBA/1 mice. The AC of the eye is injected with about 50–100 μg of CII in 10 mM acetic acid (in 5 μl; Sigma-Aldrich, St. Louis, MO, USA). Controls are the mice injected with 10 mM acetic acid alone *via* AC injection. This was followed by a subcutaneous immunization of 250 μg of CII (Sigma-Aldrich) on day 7. The CII was emulsified 1:1 in complete Freund's adjuvant (CFA; Sigma-Aldrich). Of the CII/CFA emulsion, 200 μl was injected to each animal. Either a DTH assay or a LAT assay was performed on day 14 after the AC injection of CII as explained below.

### Generation of ACAID APCs

As described previously, ACAID APCs were generated *in vitro* [7,19]. ACAID APCs from C57BL6 and DBA/1 mice were generated as described before [20]. Briefly, at concentrations of TGF-β2 that are similar to those present in the aqueous humor of the eye, APCs (2 × 10^6^ cells/ml) were cultured overnight in complete RPMI 1640 with 10 mg/ml CII and 2–5 ng/ml TGF-β2 (R&D Systems).

### *In vitro* generation of ACAID Tregs

An *in vitro* spleen cell culture system that we previously used to generate Tregs that express the same properties and surface markers as Tregs produced *in vivo* has been used [7,19]. Functional Tregs can directly inhibit DTH responses [21]. To a 100 mm Petri dish containing 5 × 10^7^ spleen cells harvested from normal C57BL6 and DBA/1 mice, CII-specific ACAID APCs (5 × 10^6^) were added and incubated for 5–7 days at 37°C before testing for the presence of Tregs.

### Immunization by subcutaneous injection

Subcutaneous injection of 250 μg of CII (Sigma-Aldrich) emulsified 1:1 in complete Freund's adjuvant (CFA; Sigma-Aldrich) was used to perform mice immunization. Each mouse received 200 μl of the CII/CFA emulsion.

### DTH assay

Delayed-type hypersensitivity responses are inhibited in association with ACAID induction [22–26]. DTH assays were performed in a similar manner as previously described [17]. Briefly, left ear pinnae were injected intradermally with ∼500 μg of CII in 20 μl. Right ear pinnae were injected with 20 μl of 10 mM acetic acid (internal control). Seven days after the subcutaneous immunization with CII/CFA, DTH assays were performed. The engineer's micrometer (Mitutoyo, Japan) was used to perform ear swelling measurements before and 24 h after CII injection. The results were measured as: specific ear swelling = (24 h measurement − 0 hr measurement) for left ear − (24 h measurement − 0 hr measurement) for right ear. Results after 48 h were calculated in a similar manner: specific ear swelling = (48 h measurement − 0 hr measurement) for left ear − (48 h measurement − 0 hr measurement) for right ear.

### LAT assay

Details of the LAT assay have been previously described [7,17,19]. Putative Tregs (1 × 10^6^ cells in 10 μl) with spleen cells (1 × 10^6^ cells in 10 μl) collected from subcutaneously immunized donors and 500 μg CII were injected to the left ear pinnae of naïve mice. After 24 and 48 h, inhibition of ear swelling responses induced by immune spleen cells was tested to confirm the presence of Tregs. The presence of Tregs will reduce ear swelling responses that are generated when antigen is added to immune spleen cells. For both the LAT and DTH assays, we used five mice per group.

### Statistics

Statistical significance was determined using Student's *t*-test. Data were expressed as mean ± SD. *P* values <0.05 were considered significant.

## Results

### Type II collagen induced ACAID in C57BL6 and DBA/1 mice *via* AC injection as confirmed by DTH assays

**Fig. 1:**
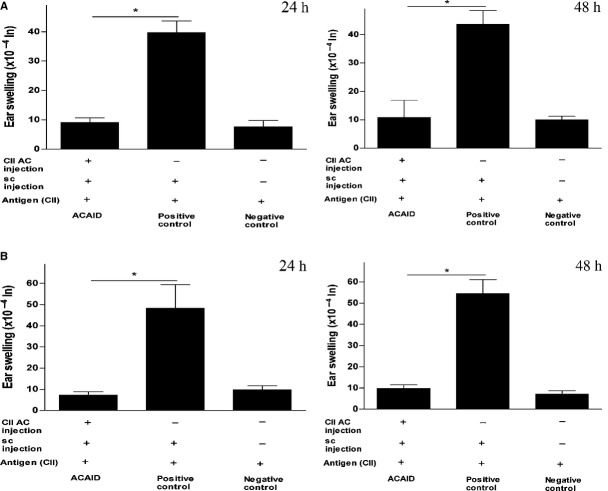
CII induces ACAID in C57BL6 and DBA/1 mice *via* AC injection as confirmed by DTH assays (**A**) CII induces ACAID in C57BL6 mice *via* AC injection. (**B**) CII induces ACAID in DBA/1 mice *via* AC injection. A DTH assay was performed as described in the materials and methods section. To induce ACAID, mice received an AC injection of CII followed by subcutaneous immunization with CII/CFA on day 7. On day 14, mice were challenged with CII (500 μg in 20 μl) intradermally in the left ear pinna, and 20 μl 10 mM acetic acid alone was injected into the right ear pinna as an internal control. Induction of ACAID was confirmed by inhibition of ear swelling responses after 24 and 48 h. The positive control mice were subcutaneously immunized with CII/CFA on day 7 and with CII on day 14, whereas the negative control mice only received the day 14 intradermal injection of CII. *P* values <0.05 were considered to be significant (*). CII, type II collagen; ACAID, anterior chamber-associated immune deviation; AC, anterior chamber; DTH, delayed-type hypersensitivity; CFA, complete Freund's adjuvant.

We have recently shown that CII could induce peripheral tolerance in Balb/c mice *via* the AC injection of CII or the intravenous injection of *in vitro*-generated CII-specific ACAID APCs [17,18]. Whether CII could induce ACAID in the arthritis-prone DBA/1 or C57BL/6 mice remains to be elucidated. We used DTH assays to test for the inhibition of CII-specific inflammatory responses. The procedure included priming the mice in the AC with CII (day 0) followed by immunizing subcutaneously with CII emulsified with CFA on day 7. This was followed with ear-testing of the AC-induced mice by intradermal injections of CII on day 14. Results showed significant inhibition of DTH responses compared to positive control mice that did not receive the AC injection, which confirmed that mice primed in AC with CII did develop peripheral tolerance (Fig. 1). This clearly shows that CII injection into the AC of DBA/1 and C57BL/6 mice could induce ACAID. The statistical significance data of experiments on C57BL/6 and DBA/1 mice was as follows: Figure 1A (C57BL/6): ACAID mice *versus* positive control, *P = 0.00009 (24 h) and *P = 0.00023 (48 h); Figure 1B (DBA/1): ACAID mice *versus* positive control, *P = 0.0035 (24 h); *P = 0.00048 (48 h).

### *In vitro*-generated Type II collagen-specific ACAID APCs induced ACAID in C57BL6 and DBA/1 mice

**Fig. 2:**
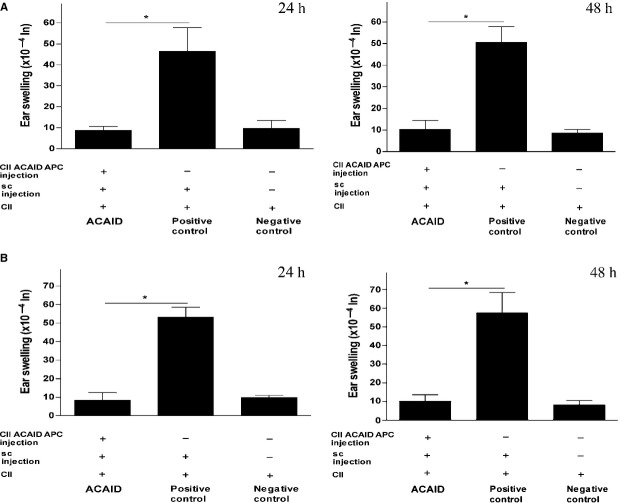
*In vitro*-generated CII-specific ACAID APCs induce ACAID in C57BL6 and DBA/1 mice as confirmed by DTH assays. (**A**) *In vitro*-generated CII-specific ACAID APCs inhibit DTH responses in C57BL6 mice. CII-specific ACAID APCs were generated *in vitro*, as described, and were adoptively transferred into C57BL6 mice (1 × 10^6^ cells/mouse). A DTH assay was performed, as described. (**B**) *In vitro*-generated CII-specific ACAID APCs inhibit DTH responses in DBA/1 mice. CII-specific ACAID APCs were generated *in vitro*, as described, and were adoptively transferred into DBA/1 mice (1 × 10^6^ cells/mouse). A DTH assay was performed, as described. Induction of ACAID was confirmed by inhibition of ear swelling responses after 24 and 48 h. *P* values <0.05 were considered to be significant (*). CII, type II collagen; ACAID, anterior chamber-associated immune deviation; DTH, delayed-type hypersensitivity; APC, antigen-presenting cell.

We adoptively transferred ACAID APCs into naïve C57BL6 and DBA/1 mice on day 0 to examine the hypothesis that *in vitro*-generated CII-specific ACAID APCs induce peripheral tolerance in C57BL6 and DBA/1 mice. The protocol for generating ACAID APCs is described in the methods section. On day 7, the recipient mice were subcutaneously immunized with CII emulsified with CFA. These mice were challenged with CII *via* intradermal injections in the ear on day 14. The significant suppression of DTH responses observed in these mice as compared with the positive control mice that did not receive ACAID APCs shows that tail vein injection of CII-specific ACAID APCs induced immune tolerance in both arthritis-prone strains of mice (Fig. 2A and B). The statistical significance data of experiments on C57BL/6 and DBA/1 mice was as follows: Figure 2A (C57BL/6): ACAID mice *versus* positive control, *P = 0.004 (24 h); *P = 0.0025 (48 h); Figure 2B (DBA/1): ACAID mice *versus* positive control, *P = 0.000012 (24 h); *P = 0.00029 (48 h).

### *In vitro*-generated ACAID Tregs impaired DTH responses

**Fig. 3:**
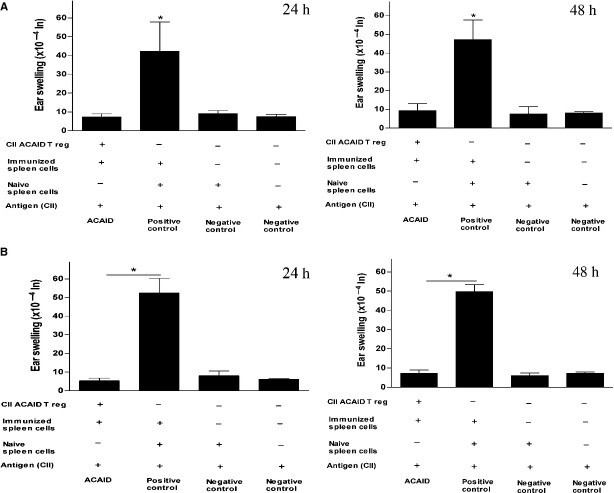
*In vitro*-generated CII-specific ACAID Tregs inhibit DTH responses in C57BL6 and DBA/1 mice as confirmed by LAT assays. (**A**) *In vitro*-generated CII-specific ACAID Tregs inhibit DTH responses in *C57BL6* mice. CII-specific ACAID Tregs were generated *in vitro*, as described, and a LAT assay was performed, as described. (**B**) *In vitro*-generated CII-specific ACAID Tregs inhibit DTH responses in DBA/1 mice. CII-specific ACAID Tregs were generated *in vitro*, as described, and a LAT assay was performed, as described. Induction of ACAID was confirmed by inhibition of ear swelling responses after 24 and 48 h. *P* values <.05 were considered to be significant (*). CII, type II collagen; ACAID, anterior chamber-associated immune deviation; DTH, delayed-type hypersensitivity; APC, antigen-presenting cell; LAT, local adoptive transfer.

We previously showed that Tregs capable of suppressing DTH responses could be generated *via* the AC injection of CII in Balb/c mice [17]. Here, we tested whether CII-specific putative Tregs generated *in vitro* could impair DTH responses. Using LAT assay, the cultures were examined for the generation of putative Tregs 5–7 days after the co-culture of *in vitro*-generated CII-specific ACAID APCs and spleen cells isolated from naïve mice. Briefly, spleen cells were isolated from naïve and CII/CFA-immunized mice. Approximately, 1 × 10^6^ CII-specific putative Tregs were mixed with an equal number of immune spleen cells (containing effector cell populations harvested from CII-immunized mice) and CII (500 μg). Of 10 mM acetic acid, 20 μl injected into the right ear pinna was used as an internal control and the suspension (20 μl) was inoculated through intradermal injection into the left ear pinna of naïve C57BL6 and DBA/1 mice. Injections of CII-immunized spleen cells and naïve spleen cells with CII constituted the positive control. The negative control received only naïve spleen cells (2 × 10^6^ cells) in addition to the CII. Impaired DTH responses to CII as compared to the positive control were demonstrated in recipients of *in vitro*-generated CII-specific putative Tregs. As expected, negative ear swelling responses have been observed in the negative control. The statistical significance data of experiments on C57BL/6 and DBA/1 mice was as follows: Figure 3A (C57BL/6): ACAID mice *versus* positive control, *P = 0.012 (24 h); *P = 0.0025 (48 h); Figure 3B (DBA/1): ACAID mice *versus* positive control, *P = 0.00034 (24 h); *P = 0.00002 (48 h).

## Discussion

Antigen entry to an immune privileged site like the eye can induce peripheral tolerance. If tolerance fails, it can trigger harmful immune responses leading to irreparable injury to terminally differentiated ocular cells encircling the corneal endothelium and cells forming the retina that lack regeneration capacity. Although ACAID-triggered peripheral tolerance could protect the complex structures encompassing the visual axis from any destructive consequences of immunogenic inflammation, blindness may ensue following damage to any of these cell types. Following antigenic entry into the eye, bone marrow-derived F4/80^+^ APCs of the iris and ciliary body pick the antigen before immigrating into the systemic circulation and homing to the spleen where they develop antigen-specific tolerance through generation of Tregs [7,8]. The suppression of DTH responses is a typical way to measure the induction of ACAID-mediated peripheral tolerance [27]. Such suppression has been shown to be mediated by antigen-specific efferent CD8^+^ Tregs [24,28,29]. Hence, it was presumed that the dominant Th2-like responses suppressed Th1 responses in ACAID.

Here, we tested the hypothesis that the AC injection of CII or the intravenous injection of CII-specific *in vitro*-generated ACAID APCs induces immune deviation and specific peripheral tolerance in recipient C57BL/6 and DBA/1 mice. Results indicated that CII-specific peripheral tolerance was generated after the AC injection. It was also generated after the intravenous injection of ACAID APCs that were CII specific. It is noteworthy that a potential application of the ACAID model is in the design of cell-based therapies administered *via* intravenous routes. Finally, we used LAT assays to test the hypothesis that *in vitro*-generated CII-specific putative Tregs induce specific peripheral tolerance. ACAID-mediated peripheral tolerance was in fact detected after the intradermal injection of the putative Tregs.

We have previously shown the induction of specific immune tolerance in Balb/c mice through injection of CII into the AC of the eye or the intravenous injection of *in vitro*-generated ACAID APCs specific to CII [17,18]. In the present study, we extended our observations to C57BL/6 and DBA/1 mice, in which arthritis could be potentially induced. These findings could be beneficial for future examinations on the effect of the induction of ACAID *via* CII on the progression of diseases in which CII is involved.

The present study is the first to report that the ability to induce CII-specific tolerance in two arthritis-prone strains of mice, namely C57BL/6 and DBA/1. It will be interesting to compare the development of arthritis in each of these two strains when CII is injected after the establishment of the disease. The findings of this study have clear therapeutic implications in RA and could also be relevant to a variety of other human diseases in which CII is involved.
